# Does Exercise Affect Telomere Length? A Systematic Review and Meta-Analysis of Randomized Controlled Trials

**DOI:** 10.3390/medicina58020242

**Published:** 2022-02-05

**Authors:** Seonghyeok Song, Eunsang Lee, Hyunjoong Kim

**Affiliations:** 1 Ez Rehabilitation Medical Center, 302 Gwanggyojungang-ro, Yongin 16943, Korea; qwerhyck@naver.com; 2 Department of Physical Therapy, Gwangju Health University, 73 Bungmun-daero 419beon-gil, Gwangju 62287, Korea; eslee@ghu.ac.kr; 3 Neuromusculoskeletal Science Laboratory, 306 Jangsin-ro, Gwangju 62287, Korea

**Keywords:** exercise, telomere, aerobic exercise, weight loss, physical activity

## Abstract

*Background and objectives:* Telomere length is an indicator of biological aging, and it shortens during cell division. A short telomere length is associated with various age-related diseases and mortality. It is suggested that physical activity has a positive effect on the rate of telomere length shortening. *Materials and Methods:* Related studies, published in electronic databases, were searched with keywords, including exercise, telomere length, and randomized controlled trial. The data were weighted and pooled through a fixed-effect model. *Results:* Of the total 49 studies searched, 7 studies with 939 participants were considered suitable, and were analyzed qualitatively and quantitatively. Exercise is beneficial to telomere length. Aerobic exercise was effective as the type of exercise (MD, −0.03; 95% CI, −0.04 to −0.01). In addition, exercise for more than 6 months, with a change in lifestyle, is beneficial for telomere length (MD, −0.02; 95% CI, −0.04 to −0.01). *Conclusions:* The type and duration of exercise for positive improvement in telomere length is aerobic exercise for more than 6 months.

## 1. Introduction

A telomere is a structure at the end of a linear chromosome, which is a repetitive nucleotide region at each end of a DNA molecule that protects the end of the chromosome and prevents degradation and fusion by maintaining genomic stability [1,2]. Telomere length shortens as cell division occurs; therefore, it is an indicator of biological aging. This is because, as a physiological process, a small part of telomeric DNA is lost during cell division [1,3].

A short telomere length is suggested to be associated with, or predict, many common aging diseases, including various geriatric diseases, such as cancer, dementia, and osteoporosis [1,4]. Furthermore, in a prospective study by Goglin et al. [5], an increase in telomere length resulted in a lower mortality rate, while a decrease in telomere length resulted in a higher mortality rate. Therefore, controlling telomere length can be a key factor in health care.

Studies of the relationship between telomeres and exercise may provide an answer as to how to control telomere length. High telomerase activity and a reduced rate of telomere attrition have been observed in endurance athletes, compared to those in inactive controls [6]. In related studies, it was reported that telomere length shortening can be reduced with moderate levels of physical activity, compared to inactivity [7]. Moreover, higher physical activity led to middle-aged twins possessing longer telomeres compared to those in inactive siblings [8]. In addition, in relation to the duration of the intervention, telomere length increased after six months of physical activity, while there was no effect in the short term [9,10].

Interest in the effects of diet and physical activity on telomere dynamics is growing, but prospective studies are lacking so far, and the effects of various exercise modalities have not been established [8,11]. However, the relationship between telomere length and exercise needs to be further investigated. Therefore, in this review, randomized controlled trials (RCTs) were synthesized from the prospective studies available to date to perform a qualitative and quantitative analysis according to the type and duration of exercise.

## 2. Materials and Methods

### 2.1. Study Design

This review followed the Preferred Reporting Items for Systematic Reviews and Meta-Analysis (PRISMA) guidelines. This review was conducted after registration in the international database of prospectively registered systematic reviews (PROSPERO, No. CRD42021278997).

### 2.2. Search Strategy and Selection of Studies

#### 2.2.1. Inclusion Criteria

1. Participants
  Participants were not classified according to specific diseases or conditions.
2. Intervention
  In the experimental group, interventions in which exercise was the main focus were selected.
3. Comparisons
  Inactivity or usual care was selected for comparison with interventions that included exercise.
4. Outcomes
  In our review, only the effect on telomere length was investigated.
5. Types of studies
  Only RCT retrieved through international electronic databases were included.

#### 2.2.2. Exclusion Criteria

The exclusion criteria are as follows: non-human studies, intervention refers to lifestyle or physical activity, not a specific exercise, retrospective study, and not RCT.

#### 2.2.3. Literature-Search Strategy

Data were collected between October and November 2021. Two researchers (H.K. and S.S.) with experience in systematic reviews and meta-analyses performed an independent search. The search was performed by combining exercise, telomere length, and RCT. Index terms and identified keywords were retrieved from the following international electronic databases: Medical Literature Analysis and Retrieval System Online (MEDLINE), Excerpta Medica dataBASE (EMBASE), Cumulative Index to Nursing and Allied Health Literature (CINAHL), and Physiotherapy Evidence Database (PEDro). Additional data searches were performed with index terms and keywords identified on Google Scholar.

#### 2.2.4. Study Selection and Data Extraction

For studies that were searched for in the four international electronic databases, duplicate data were removed using a reference manager (EndNote 20, Thomson Reuters, NY, USA). Screening was conducted according to the inclusion criteria by two researchers (H.K. and S.S.), by checking the title and abstract. The researchers then discussed the reasons for their exclusion. Finally, the selected studies were classified, and features were extracted. All selection and extraction of data retrieved from the database were independently performed by two researchers.

#### 2.2.5. Quality Assessment

The quality assessment of the studies selected in this review was evaluated using the seven-item Cochrane RoB tool developed by the Cochrane Bias Method Group. RoB was rated as low (+), uncertain (?), or high (−) by two researchers with meta-analysis research experience. In the same way, for study selection and data extraction, if there were items that did not match, the original text was reviewed and re-evaluated.

### 2.3. Strategy for Data Synthesis

Data synthesis was analyzed using RevMan 5.4 (The Cochrane Collaboration, Oxford, England), an editing review software developed by Cochrane. Meta-analysis was performed when there were quantitative values of the same outcome variable, or post-test outcome variable, and was performed when there were three or more studies for each outcome variable. Quantitative analysis was performed using the mean difference representing the change from baseline. Fundamentally, it was expressed as a fixed-effects model, but when the heterogeneity was judged to be high, a random-effects model was used. The heterogeneity of the selected RCTs was confirmed using the chi-square test and I^2^ test provided by Cochrane. If the I^2^ value is less than 40%, this indicates low heterogeneity, 50–75% indicates significant heterogeneity, and more than 75% indicates high heterogeneity [12]. Publication bias of the studies was analyzed using funnel plots [13].

## 3. Results

This review was registered with PROSPERO in October 2021, and data synthesis was completed on 10 December 2021.

### 3.1. Literature Search and Characteristics of the Included Randomized Clinical Trials

**Figure 1:**
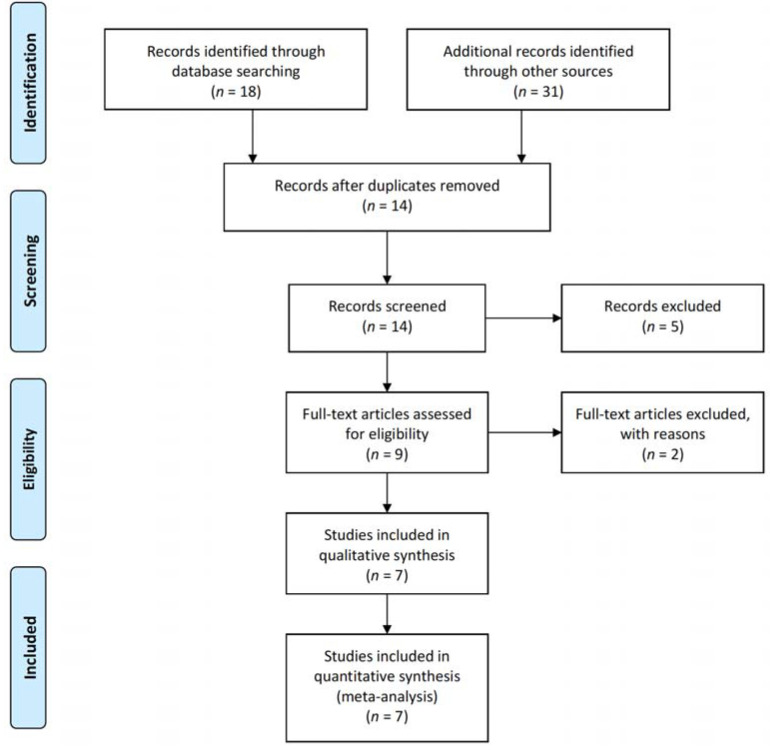
PRISMA flow diagram.

A total of 18 papers were searched for using the international electronic databases, and 31 papers were added through additional searches, resulting in a total of 49 papers. For the processing of duplicate data, 14 papers were excluded through EndNote 20. Five papers were excluded by reviewing the titles and abstracts, according to the inclusion criteria determined by two researchers. Finally, two papers were identified through the title and abstract, but the results were not provided as numerical values, so they were excluded and seven papers were selected [10,14,15,16,17,18,19]. The seven selected papers were analyzed through a systematic review and meta-analysis (Figure 1).

### 3.2. Methodological Quality Assessment

**Figure 2:**
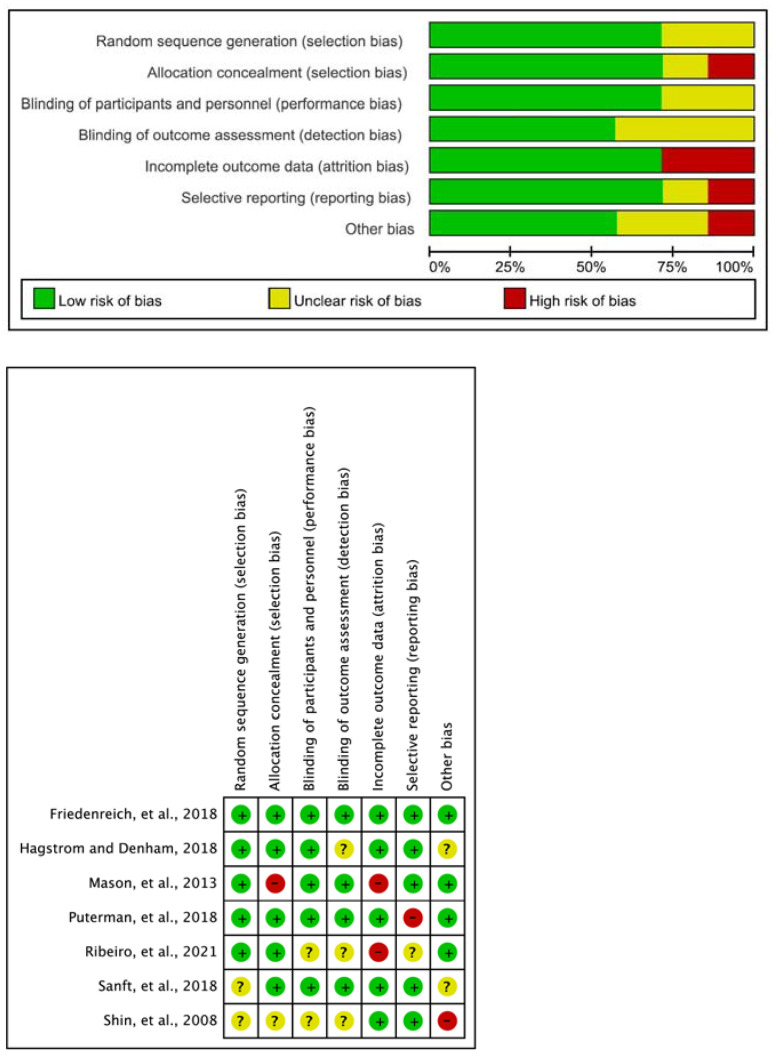
Risk of bias of the systematic review.

Two researchers conducted a pilot test on three studies in advance for a more objective quality assessment. The methodological quality assessments of the seven experimental studies included the following: random sequence generation (uncertainty: 2, low: 5), allocation concealment (high: 1, uncertain: 1, low: 5), and the blinding of participants and personnel (uncertainty: 2, low: 5). A blinded outcome assessment was also conducted (uncertainty: 3, low: 4), which assessed incomplete outcome data (high: 2, low: 5), selective reporting (high: 1, uncertain: 1, low: 5), and other biases (high: 1, uncertain: 2, low: 4) (Figure 2).

### 3.3. Exercise and Telomere Length

For review, 939 participants whose telomere length was confirmed through each of the various exercises in the 7 RCTs were included. Only exercise, not lifestyle or physical activity changes, was included. The only outcome measured was telomere length. The seven selected studies are shown in Table 1.

### 3.4. Types of Exercise That Affect Telomere Length

**Figure 3:**
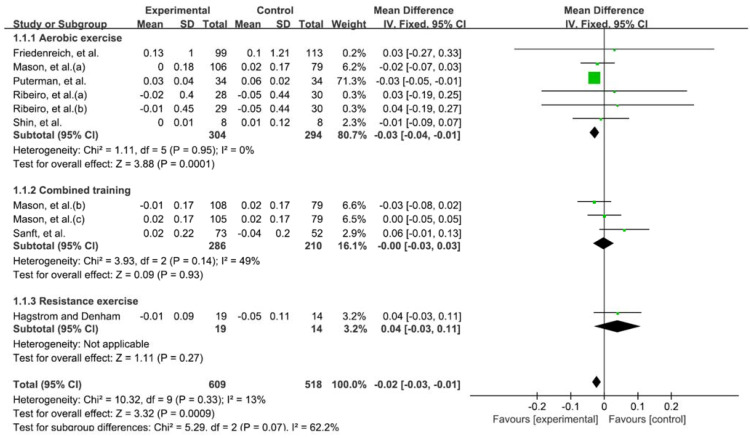
Forest plot on the effect of exercise on telomere length. Mason et al. (a) aerobic exercise; (b) aerobic exercise and combinded training; (c) combinded training. Ribeiro et al. (a) continuous aerobic training; (b) intermittent aerobic training.

Telomere length was evaluated in 939 patients from 7 RCTs. Compared to the control group (usual care or inactivity), significant improvement was observed in the experimental groups that performed exercise. The results analyzed through the fixed-effects model are −0.02; 95% confidence interval (CI), −0.03 to −0.01; heterogeneity (χ^2^ = 10.32, df = 9, I^2^ = 13%); overall effect (Z = 3.32, *p* < 0.001). In the subgroup analysis classified by exercise type, combinded training and resistance exercise did not show significant improvement compared to the control group, and only aerobic exercise showed significant improvement (−0.03; 95% CI, −0.04 to −0.01; heterogeneity (χ^2^ = 1.11, df = 5, I^2^ = 0%); overall effect (Z = 3.88, *p* < 0.001)) (Figure 3).

### 3.5. Telomere Length According to Duration of Exercise

**Figure 4:**
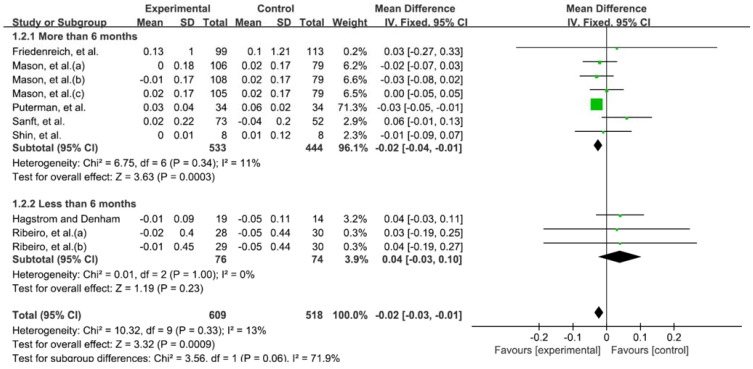
Forest plot on the effect of telomere length according to the duration of exercise. Mason et al. (a) aerobic exercise; (b) aerobic exercise and combinded training; (c) combinded training. Ribeiro et al. (a) continuous aerobic training; (b) intermittent aerobic training.

The quantitative comparison according to the duration of exercise was classified into 6 months or more, and less than 6 months. When the type of exercise was not distinguished, exercise for more than 6 months had a significant effect on telomere length (−0.02; 95% CI, −0.04 to −0.01; heterogeneity (χ^2^ = 6.75, df = 6, I^2^ = 11%); overall effect (Z = 3.63, *p* < 0.001)) (Figure 4).

### 3.6. Publication Bias

**Figure 5:**
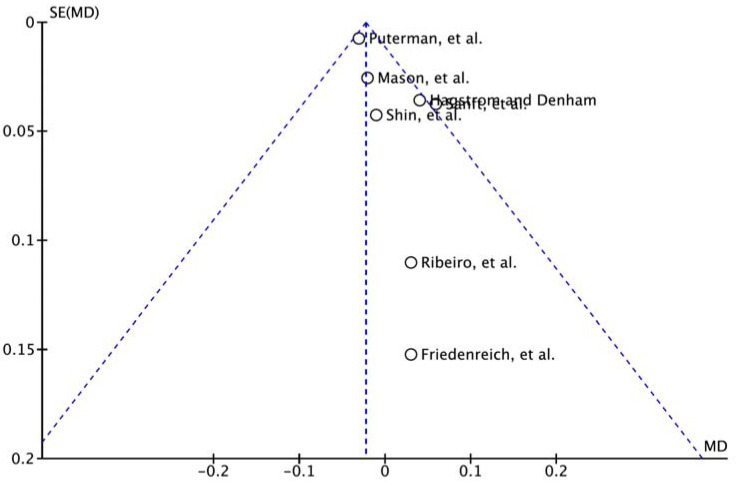
Funnel plot of comparison.

Seven studies were included, and funnel plots were created as a visual assessment to evaluate potential publication bias. Although it is not clear because it is a visual evaluation, it is not symmetrical and is skewed to the right, so it can be observed that there is a slight risk of publication bias (Figure 5). However, the study protocol was approved in advance as a prospective study in six studies, except for one case that was not identified in seven studies.

## 4. Discussion

In our review, seven RCTs were selected, and qualitative and quantitative analyses were performed. Classification by type and duration of exercise was integrated using the effect size. A total of 939 participants participated in this study. Overall, compared to usual care or inactivity, aerobic exercise for six months or more had a significant effect on the rate of telomere length shortening. These results are considered to have contributed, in part, to the clinical demonstration of the association between exercise and telomere length.

Given that we only analyzed RCTs, systematic reviews and meta-analyses were performed on seven studies. Naturally, there were studies that did not show any significant change, but, as a result, aerobic exercise showed a positive association with the type of exercise (Z = 3.88, *p* < 0.001), and a positive association with the duration of exercise for more than six months (Z = 3.63, *p* < 0.001). In most of the studies, the sample size was large and the quality evaluation through risk of bias was also excellent.

In previous systematic review studies, it was reported that exercise had a potential effect on telomeres and that moderate-intensity exercise would be beneficial [8]. In addition to these results, other systematic reviews confirmed that telomere length was longer in active people than in inactive people, regardless of exercise intensity [20]. Taken together, it may be said that aerobic exercise with moderate intensity in active conditions is more effective than in inactive conditions. In addition, in a study that predicted mortality through telomere length in duration of exercise, an increase over five years reduced the mortality rate [5]. To make this clearer, it has been suggested that comprehensive lifestyle changes, including exercise for five years, increase telomere length [21]. Based on these results, it was judged that there would be differences according to the period. In this review, subgroup analysis was classified based on six months of data, in order to quantitatively perform subgroup analysis in seven RCTs. The results were different, and when the type of exercise was not distinguished, and the exercise was performed continuously for more than six months, there was a significant effect on telomere length compared to the control group.

In studies of lifestyle changes, the intervention period is mainly reported on the basis of six months [22,23,24]. Therefore, it is thought that regular activity over a long period of time, changes in physical activity, [8] and the intensity of physical activity [20] will significantly affect telomeres. Although the effects of different types of exercise on telomere length have not yet been classified, various trials are being conducted. According to the results of the quantitative analysis in this meta-analysis, aerobic exercise was effective. Aerobic exercise has a beneficial effect in the prevention of vascular diseases, such as arterial stiffness. This is a result of a reduction in oxidative stress [25]. This suggests that telomere length is also positively related, due to its effect on telomerase activation, by enhancing the maximal oxygen uptake [26]. It was also reported that aerobic exercise had a positive effect on telomere length in studies not synthesized in this review [27,28,29]. The current evidence on the relationship between exercise and telomere length shortening reports that long-term exercise improves antioxidant activity and helps REDOX balance [30,31]. In addition, exercise has been reported to improve inflammatory balance through a reduction in C-reactive protein, interleukin−6, and tumor necrosis factor α (TNFα) levels [32].

Taken together, our systematic review and meta-analysis of RCT find that, at a recommended intensity, exercise for more than six months can positively change telomere length. In addition, aerobic exercise more positively affects telomere length than other types and intensities of exercise. However, it is difficult to justify these results with only seven RCTs. In addition, it is thought that there was publication bias through use of the funnel plot. Nevertheless, given that only RCTs compared to the control group were included under equivalent conditions, there would be an unknown potential effect on the effect of exercise on telomere length. As a limitation of this review, no comparison was made according to specific disease groups, and the effect of age was not addressed. In further studies, RCTs with various strengths and types will be required, as will quantitative analysis by classification according to each disease group or age.

## 5. Conclusions

Exercise has a beneficial effect on telomere length compared with usual care or inactivity. Exercise for more than six months is associated with changes in telomere length. The evidence gathered to date shows that aerobic exercise slows the decline in telomere length. However, further studies are needed to determine the effect of various ages, diseases, or exercise intensities on telomere length. 

## Figures and Tables

**Table 1:** Characteristics of included studies.

Study	Subject	Sample Size	Duration	Exercise Intensity	Control	Author’s Conclusion	Country, Setting
Friedenreich, et al., 2018	50–74 years old	AG = 99 CG = 113	12 months	Aerobic exercise five days/week (supervised three days/week), 45 min/session, achieving 70–80% heart rate reserve.	Usual inactivity	One year of aerobic exercise did not significantly change telomere attrition in healthy postmenopausal women.	Canada, Westside Recreation Centre in Calgary
Hagstrom and Denham, 2018	Breast cancer	RG = 19 CG = 14	16 weeks	Supervised RT was performed three times per week for 60 min per session. The exercise load was set at an 8 repetition maximum.	Usual inactivity	Resistance training is a safe intervention that does not accelerate biological aging.	Australia, University of Western Sydney exercise science laboratories
Mason, et al., 2013	Postmenopausal women	AG = 106 AWG = 108 WG = 105 CG = 79	12 months	*- Combined training:* total daily energy intake of 1200–2000 kcal/day based on baseline weight, <30% daily energy intake from fat, and a 10% reduction in body weight by 6 months with maintenance thereafter to 12 months. *- Aerobic exercise:* ≥45 min of moderate-to-vigorous intensity exercise, 5 days/week (225 min/week) for 12 months	Four group nutrition classes and 8 weeks of facility exercise training with individualized guidance from an exercise physiologist	Twelve months of dietary weight loss and aerobic exercise did not change telomere length in postmenopausal women.	USA, Fred Hutchinson Cancer Research Center
Puterman, et al., 2018	Dementia caregivers	AG = 34 CG = 34	24 weeks	An exercise program starting with a self-selected activity 3 times of 20 min each week and increasing to 4 repetitions per week.	Received free gym memberships and a similar personalized fitness program	Aerobic exercise to improve health indicators and attenuate cellular aging in high-risk samples.	USA, Clinical & Translational Science Institute Clinical Research Services
Ribeiro, et al., 2021	Polycystic ovary syndrome	CAI = 28 IAT = 29 CG = 30	4 months	It lasts evenly and gradually from 30 min in the first week to 50 min in the last week. Target strength training areas followed ACSM recommendations.	Maintaining daily physical activity	Booth exercises reduced obesity indices and hyperandrogenism on PCOS women without changes in telomere length or inflammatory biomarkers.	Brazil, Ribeirão Preto Medical School-University of São Paulo
Sanft, et al., 2018	Breast cancer	WG = 73 CG = 52	6 months	Reducing calories to 1200–2000 kcal/day, adjusting to baseline body weight, and reducing dietary fat to less than 25% of total energy intake	Brochures on nutrition and physical activity are available	Suggests that weight loss interventions may prolong telomere length compared to shortening in usual care counterparts.	USA, Yale-New Haven Hospital
Shin, et al., 2008	Obese middle-aged women	AG = 8 CG = 8	6 months	Three days a week for 6 months. Each session consisted of 10 min of warming up, 45 min of treadmill walking/running, and 5 min of cooling down.	Usual inactivity	The lengths of telomere in leukocytes were not influenced by both mid-intensity and high intensity of exercise stress.	Republic of Korea, Seoul national university

AG: aerobic exercise group, AWG: aerobic plus combinded training group, CAT: continuous aerobic training, CG: control group, IAT: intermittent aerobic training, RG: resistance exercise group, WG: combinded training group.

## Data Availability

Not applicable.
